# Managing maternity: Moving care, not patients, using artificial intelligence (AI), internet‐of‐things (IOT) and point‐of‐care testing (POCT) devices

**DOI:** 10.1002/ijgo.70942

**Published:** 2026-03-17

**Authors:** Lin Foo, Mahesh Choolani, Aris Papageorghiou, Hema Divakar, Hassan Shehata, Nir Melamed, Gabriel Jones, Vyta Senikas, Eline M. Van der Beek, Nandita Palshetkar, Gabrielle Saccone, Vincenzo Berghella, Justin Konje, Bhaskar Bhatt, Augusto Cam, Moses Obimbo, Anne Beatrice Kihara, Moshe Hod

**Affiliations:** ^1^ Department of Obstetrics & Gynaecology National University Hospital Singapore; ^2^ Department of Obstetrics and Gynaecology, Yong Loo Lin School of Medicine National University of Singapore Singapore; ^3^ Department of Obstetrics and Gynaecology, National University Centre for Women and Children National University Health System Singapore; ^4^ Nuffield Department of Women's & Reproductive Health Oxford Maternal and Perinatal Health Institute (OMPHI), University of Oxford Oxford UK; ^5^ Divakars Specialty Hospital Bengaluru India; ^6^ Epsom and St Helier University Hospitals NHS Trust London UK; ^7^ Centre for Reproductive Immunology and Pregnancy Surrey UK; ^8^ Royal College of Obstetricians and Gynaecologists London UK; ^9^ Sunnybrook Health Sciences Centre, Obstetrics & Gynaecology University of Toronto Toronto Ontario Canada; ^10^ Oxford Digital Health Labs, Nuffield Department of Women's and Reproductive Health University of Oxford Oxford UK; ^11^ Faculty of Medicine McGill University Montreal Quebec Canada; ^12^ Department of Pediatrics, University Medical Centre Groningen University of Groningen Groningen The Netherlands; ^13^ Nestle Institute of Health Sciences, Nestle Research Lausanne Switzerland; ^14^ Lilavati Hospital Mumbai India; ^15^ Department of Neuroscience, Reproductive Science Ad Dentistry, School of Medicine University of Naples Federico II Naples Italy; ^16^ Department of Maternal‐Fetal Medicine, Department of Obstetrics and Gynecology Thomas Jefferson University Philadelphia Pennsylvania USA; ^17^ Feto‐Maternal Centre Doha Qatar; ^18^ Department of Obstetrics & Gynaecology Weill Cornell Medicine Doha Qatar; ^19^ School of Design, UPES Dehradun India; ^20^ Universidad Peruana de Ciencias Aplicadas Lima Peru; ^21^ Department of Human Anatomy & Medical Physiology University of Nairobi Nairobi Kenya; ^22^ Department of Obstetrics & Gynaecology University of Nairobi Nairobi Kenya; ^23^ Sackler Faculty of Medicine, Mor Comprehensive Women's Health Care Center Tel Aviv University Tel‐Aviv Israel

**Keywords:** artificial intelligence, deep learning, digital health, FemTech, hybrid clinics, large language models, machine learning, maternity care

## Abstract

The integration of artificial intelligence (AI) into healthcare is accelerating and maternity care is at a pivotal moment for the strategic implementation of these technologies. This article explores how AI‐assisted women's health innovations, often termed “FemTech,” may transform pregnancy care by addressing long‐standing disparities: enhancing diagnostic precision and supporting the obstetric workforce. We outline three domains in which AI is poised to drive change: where women are cared for, how they are cared for, and who delivers their care. First, decentralized AI combined with Internet of Medical Things (IoMT) devices can extend prenatal monitoring into homes, reducing reliance on clinic visits and expanding access for underserved populations. Second, predictive and reinforcement learning algorithms enable personalized, adaptive care across the reproductive continuum, from preconception to postpartum, moving beyond static risk models and uniform treatment approaches. Third, AI has the potential to augment the maternity workforce by offering generative tools for patient engagement, clinical decision support and automation of ultrasound imaging, while ensuring clinician oversight remains central. Future adoption will depend on global economic and geopolitical dynamics, with the USA and China currently leading in patents, publications, and model development. Equitable integration will require explainable AI, transparent validation, multinational benchmark datasets, and robust governance on safety and consent. Ultimately, AI‐powered technologies should complement, not replace human expertise, embedding digital innovation within a model of maternity care that preserves empathy and clinical judgment.

## 1 INTRODUCTION

Artificial intelligence (AI) has become a material feature of digital health and clinical workflows. The advent of AI, colloquially known as the “Digital Age,” is predicted to impact on the healthcare industry in such a profound manner as to set it apart from technologies of the “Information Age” that came before.

AI systems are engineered statistical models trained on data rather than living cognition. They are already embedded in healthcare workflows: supporting diagnostic triage and enabling remote monitoring. In obstetrics and gynecology, practical deployments include rapid imaging support and algorithm‐aided decision tools in routine pathways. Yet, even as these innovations gain ground, longstanding disparities of healthcare outcomes remain. If AI technology evolves quickly but the structures around it do not, the healthcare gap might widen between those reached by the future, and those still awaiting its arrival.

The American College of Obstetricians and Gynecologists and the International Federation of Gynecology and Obstetrics have emphasized a rethink of pregnancy care to close equity gaps.^1^, ^2^ We propose that AI‐powered women's health provides a practical bridge between “blue sky” industry innovation and clinical application. These technologies are often referred to as “FemTech” (female technology), and includes software, hardware, wearables, Internet of Medical Things (IoMT) and smartphone applications that offer digital solutions for women's health.^3^

The integration of AI‐enabled women's health technologies into mainstream health provision faces substantial barriers. First, this sector is chronically underfunded; only 2% of venture capital funding was allocated to women's health start‐ups in 2019.^4^ However, it is predicted that by 2030 the women's health market will be valued upwards of USD1.2 trillion with a pipeline of catered devices and platforms.^5^, ^6^ Second, AI systems are heavily reliant on the quality of training datasets. These have gender biases stemming from historical exclusion of pregnant women from medical trials. These problems can be mitigated by actively curating large, high‐quality training datasets, e.g. the Oxford Maternity Dataset.^7^

**FIGURE 1:**
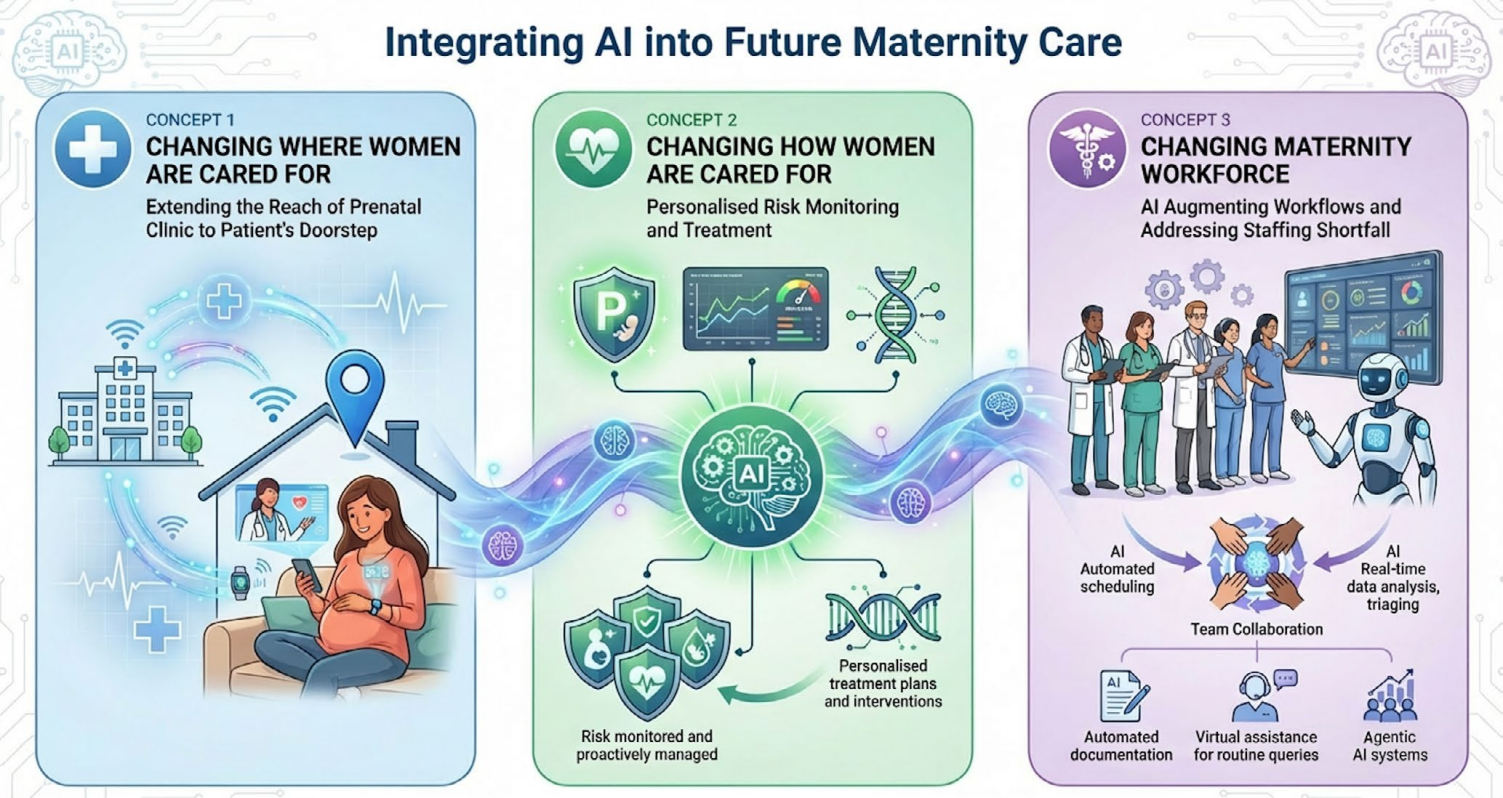
Three conceptual approaches to future AI‐allied maternity care.

Ultimately the value is not to be found in AI models themselves, but in our abilities to harness them. This article presents three main concepts that will shape the landscape of AI‐allied pregnancy care: changing where women are cared for, how they are cared for, and who delivers their care (Figure 1). We also outline anticipated future considerations to inform strategies in leveraging AI for pregnancy care.

## 2 CONCEPT 1: CHANGING WHERE WOMEN ARE CARED FOR

### 2.1 Bringing the clinic to the patient, rather than the patient to the clinic

The standard format of pregnancy monitoring consists of regular face‐to‐face visits for all patients. This rigid schedule falls short of addressing social and structural health determinants and a growing proportion of patients who do not access the full schedule of pregnancy care.^8^, ^9^ A forced change to this exacting schedule occurred during the COVID‐19 pandemic. There was a notable growth of telemedicine and shifting attitudes of traditional physician–patient relationships, with an emphasis on remote healthcare.^10^, ^11^ Post‐COVID telemedicine has not been widely adopted in obstetrics due to lack of directed strategies. Most telemedicine ventures were rolled out on a “stand‐alone” basis, e.g. blood pressure monitoring, while other pregnancy care remained via the traditional route. This increases complexity of teams interacting with patients and paradoxically reduces continuity of care.

**FIGURE 2:**
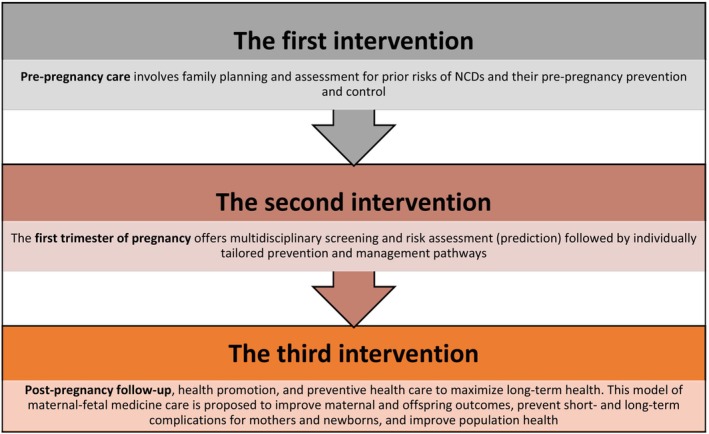
The PregCare model based on a triple intervention multidisciplinary approach to maternal and fetal medicine (reprinted from Hod et al.^1^). NCD, non‐communicable disease.

Recently, Hod et al. outlined a “PregCare” framework that leverages AI for pregnancy care.^1^ This three‐level intervention model (Figure 2) is predicted to halve the frequency of patient visits using point‐of‐care (POC) devices and virtual caregiver interactions via a central base. A key feature in this model is AI‐enabled IoMT, next‐generation bio‐analytical tools that combine network‐linked devices (wearables, POC devices) with healthcare software.

Simple IoMT applications easily transferrable to prenatal care include POC measures of ferritin^12^ (e.g. IronScan™) and glucose levels. Home blood glucose monitoring is commonplace in diabetes management but enhancing current models with AI can advance utility in the following ways. First, most POC platforms have limited multiplexing capabilities thus restricting applications for patients with co‐morbidities. Furthermore, subjective interpretation of results, such as determining whether a faint test line indicates positive or negative results, complicates clinical deployment.^13^ Integrating AI into POC can address these limitations by combining advanced sensing modalities and repeated readouts with machine‐learning (ML) algorithms that improve quantitative interpretation.^14^

The next natural progression would be IoMT devices that provide automation of management. Differing from the “PregCare” model above, this involves decentralizing AI connections and considering a cloud‐based approach. In a decentralized system, POC device would detect anomalies and then integrate this with previously collected data, the output of which is classified by a computerized rule‐based system, allowing stratification to relevant workflows.

The final evolvement would be incorporation of the newest facet of AI: agentic AI. Differing from rules‐based AI, which is narrowly task‐specific, agentic devices can act autonomously without the need for constant human guidance. Agentic AI relies on a complex ensemble of ML, natural language processing, and automation technologies.^15^ These models process sequences much like a clinician would, independently searching databases or triggering workflows to complete activities. With enriched reasoning and execution capabilities, they have a greater ability to sift information sources for quality and reduce inaccurate outputs. However, users of agentic AI must be careful to properly constrain resources available to the agent. Ultimately, these technologies reduce reliance on centralized cloud‐based and human resource infrastructures. By improving accessibility for patients, it extends the reach of the prenatal clinic right to the patient's doorstep (Box 1).

BOX 1 **Reimagining the reach of prenatal care.**

AI‐empowered digital advances potentially offer scalable patient‐centered pathways to bridge gaps in maternal health, particularly in underserved remote populations. AI‐powered IoMT pregnancy care:

- Shifts locus of care from clinic to home.
- Addresses long‐standing barriers to healthcare access.
- Empowers patients in low‐ and middle‐income countries to engage in self‐monitoring and timely intervention.

## 3 CONCEPT 2: CHANGING HOW WOMEN ARE CARED FOR

### 3.1 The right care for the right patient

#### 3.1.1 Personalized prenatal monitoring

Traditional methods of pregnancy risk assessment fall short of capturing the complex and dynamic nature of gestation specific conditions and compound risks. Predictive AI (PA) algorithms can better identify patterns within medical data for prediction of future complications. As these algorithms continuously learn from new data, predictive accuracy improves over time.^16^

In obstetrics, PA‐based apps have been utilized in pre‐term birth (QUIPP^17^) and placental function risk stratification (Tommy's App^18^). These apps are focused on identification of an “endpoint,” i.e. the occurrence of a disease during pregnancy and their application stops after the occurrence of the endpoint. Future AI tech can build on this in the following ways.

First, rather than being limited to predicting a disease endpoint, AI solutions can be utilized to project subsequent disease progression and treatment response. This can be achieved with ML calculations of probabilities of natural progression. By producing anticipated trajectories of disease course through pregnancy, ML could facilitate personalized monitoring plans and offer more optimized timing of intervention (or abstention thereof). Further, ML layered with reinforcement learning techniques can adapt management plans once a complication has occurred and collaborative filtering techniques can consider grouped patient preferences by analyzing data from similar patients.

In non‐pregnant cohorts, ML techniques such as Multilayer Perceptron and Bag Decision Trees have demonstrated success in predicting the need for dialysis in chronic kidney disease^19^ and progression of diabetes.^20^ These existing models cannot be simply adopted in obstetrics due to gestation‐specific physiology that alter disease course over a relatively short period of time. Therefore, pregnancy‐specific ML datasets are required, ideally longitudinal designs encompassing pre‐or early‐conception to postpartum periods to truly pregnancy disease course. ML models should be validated with “out of sample testing” against benchmark data. Clinicians should understand the populations from which ML algorithms were drawn, as well as the likelihood of a false positive. The latter arises if ML overanalyzes data (“over‐fitting the graph”), thereby finding patterns in randomness. Equally important is ensuring that the ethnic, cultural, and socioeconomic composition of training populations reflects the diversity of those who will ultimately receive care.

**FIGURE 3:**
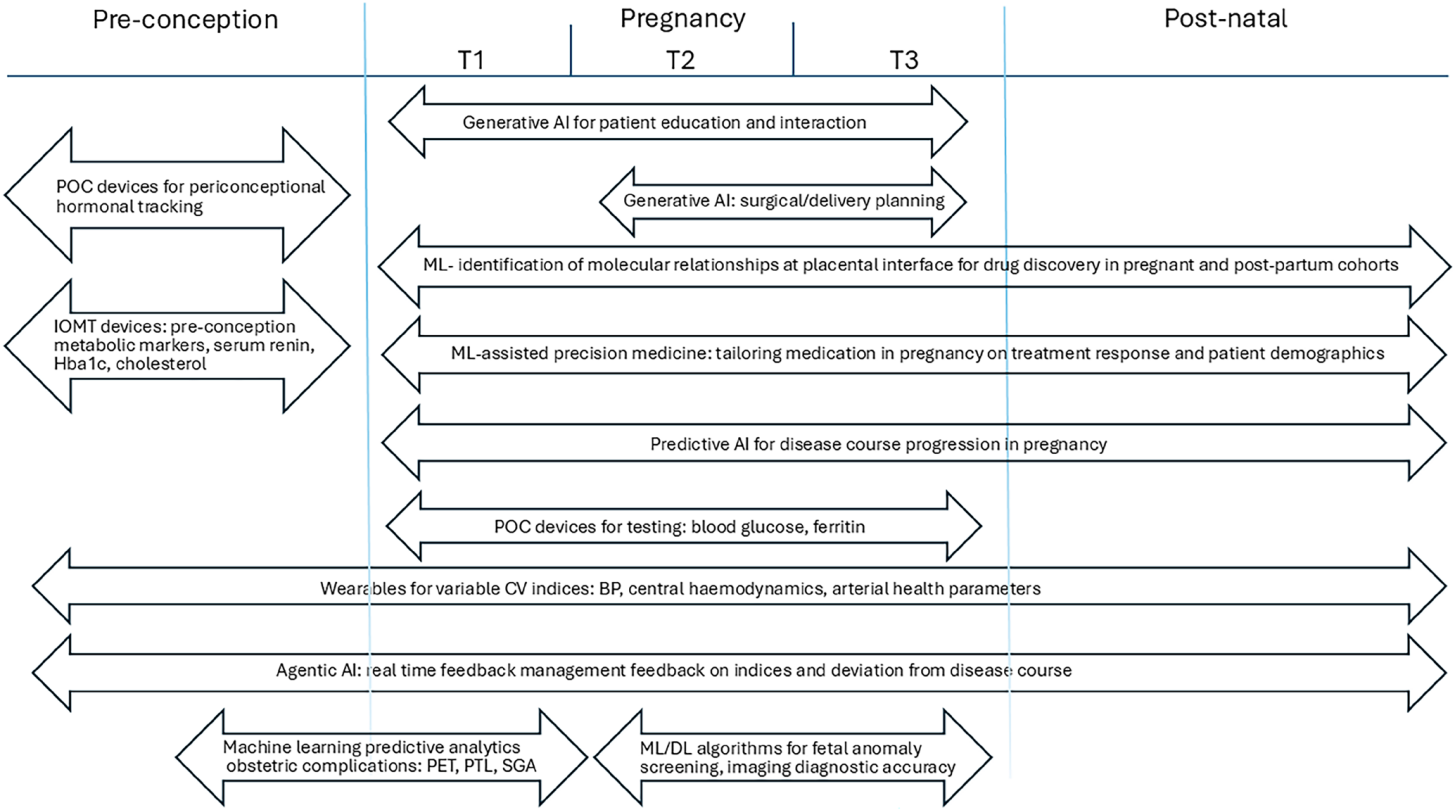
Applications of AI‐powered women's healthcare technology from pre‐conception through to the postpartum period. BP, blood pressure; CV, cardiovascular; DL, deep learning; IOMT, Internet of Medical Things; ML, machine learning; PET, pre‐eclampsia; POC, point of care; PTL, preterm labor; SGA, small for gestational age.

ML‐assisted monitoring strategies could also be applied to the epochs that lie outside pregnancy but are equally critical: the pre‐conception and postpartum period (Figure 3). Pre‐conception health is strongly associated with gestational manifestation of conditions such as diabetes and hypertensive disease.^21^, ^22^ This period remains largely underexplored due to a lack of data of women of child‐bearing age whom, as a demographic, rarely require medical visits before pregnancy. This can be resolved with AI in the form of home‐testing based biosensors. An example is tracking of peri‐conception biomarkers, such as luteinizing hormone and human chorionic gonadotropin levels, using implantable biosensors.^23^ This provides more accurate estimations of ovulation and implantation intervals and allows clinicians to facilitate timely pregnancy visits or anticipate complications such as spontaneous pregnancy loss.^24^ This concept can also be applied to track pre‐pregnancy cholesterol or renin levels to predict manifestation of metabolic diseases in pregnancy. Accruing data on pre‐conception and antenatal events into a digitalized pregnancy passport^25^ would be a useful adjunct for postpartum intervention and monitoring programs, with the aim of reducing risk of future health events.

#### 3.1.2 Personalized treatments for mothers

The convergence of AI and precision medicine leverages sophisticated computation to recommend treatments that are most effective based on unique patient attributes.

A promising avenue to implement this is in the management of pre‐eclampsia. Current pharmacological approaches to pre‐eclampsia are a “one‐size‐fits‐all” strategy, with first‐line treatments usually being a choice between beta‐blockers and/or calcium channel antagonists. There is mounting recognition of two differing phenotypes of pre‐eclampsia that are distinguishable by the patterns of cardiovascular imbalances.^26^ Ideally, the choice of hypotensive agent will be guided by non‐invasive cardiovascular monitoring,^27^, ^28^, ^29^ including blood pressure (BP), systemic vascular resistance, and arterial tone.^30^ The main drawbacks are additional hospital visits for cardiovascular testing that are, at present, largely laboratory based. Furthermore, these tests capture “snapshots” of parameters, e.g. BP that are by nature fluctuant’;^31^ therefore, one‐off readings are unlikely representative of true discordance.

A solution could come from AI‐powered FemTech in the form of non‐invasive wearables. These include commercial smartwatches, bands, and rings commonly used by patients in everyday life that analyze continuous parameters, including step count, heart rate and rhythm, BP, and maximum oxygen uptake (VO_2_ max), with high accuracy.^32^ This provides a more comprehensive view of hemodynamic changes and are “in‐field,” i.e. captured during everyday activity, which is more reflective of true trends. An unexpected benefit of wearable monitoring is the positive effect on patient behavior. Studies have shown better BP control and adherence to medication with remote monitoring.^33^ The boom of wearables now extends to more sophisticated cardiovascular parameters. A recent example is the utilization of a smart ring AI‐based advisor (GONDOR‐AS, Clinical Trials ID: NCT06644014) to record and reduce pulse‐wave velocity, an index of arterial health, abnormalities of which are strongly associated with pre‐eclampsia.^34^

When interpreting continuous data from wearable devices, substantial noise arises from motion artifacts as well as intra‐ and inter‐subject variability. Techniques such as ML‐based representation learning can improve the ability of ML models to extract meaningful information from raw, multidimensional, and unstructured data.^35^

Overall, the integration of AI solutions into personalized medicine in obstetrics may help move beyond the traditional “trial and error” approach, shifting the focus from reactive management to prevention. With the right implementation, these technologies have the potential to accelerate improvements in pregnancy outcomes beyond what has previously been seen (Box 2).

BOX 2 **Emphasis on personalized, continuous, and tech‐enabled obstetric care across reproductive timeline.**

- Static vs. dynamic risk assessment; need for longitudinal monitoring.
- Expanding AI‐enabled tech to pre‐conception and postpartum.
- Precision treatment with AI.

## 4 CONCEPT 3: CHANGING THE WORKFORCE THAT CARES FOR WOMEN

### 4.1 Our new digital colleagues

The maternity workforce consists of obstetricians and allied health professionals (midwives, laboratory staff, community attendants, sonographers, anesthetists, theater personnel). Clinicians approach a future of parallel working with AI and inevitably the face of the workforce will change to accommodate this. A clear benefit of AI‐assisted care is addressing the shortfall of healthcare workers, predicted to be 18 million workers by 2030.^36^ A mistake would be to integrate AI solutions in circumstances where they are bound to fail, such as situations that require empathy, an attribute that does not yet have a translatable analog in machines. The following section outlines examples of how, in appropriate settings, AI‐enabled women's health technology can enhance the obstetric workforce.

#### 4.1.1 Generative AI for patient engagement and education

By combining vision and language models, generative AI enables personalized multimedia content generation through natural language interactions and images.

An example of application in obstetrics could be the development of a “visual partogram.” Patients in labor often find it difficult to decipher descriptions of cervical dilatation, fetal station, and position. By inputting partogram parameters into an AI interface that links with a phone app or an in‐built receiver screen in the labor room, simulated videos of labor progress could be produced for each patient. This could be applied when counseling for intervention, e.g. a rotational delivery. AI‐generated video aids could also be formatted with captions in the patient's native language. Generative AI creates voice‐based interfaces that recognize different languages in low‐ and middle‐income countries (LMICs), allowing patient interactions with voice commands.^37^ This is particularly beneficial for patients with lower literacy levels.

#### 4.1.2 Generative AI for better understanding of pathology

In cardiology, generative AI systems have created simulations of cardiac catheterizations that help with procedural planning and training.^38^ This is translatable to more advanced obstetric surgeries, such as for abnormally invasive placentas (AIPs). Designs for AI‐based surgical support systems could combine two‐dimensional imaging data from ultrasound and MRI into navigable three‐dimensional models to improving surgical planning and precision. Further steps include exploring the potential of AI to predict the depth of visceral placental invasion using deep learning (DL). DL techniques have been utilized to predict depth of sub‐mucosal invasion in colorectal cancer with a positive predictive value of 84.4%.^39^

On a day‐to‐day basis, AI could be integrated into antenatal clinics, particularly in remote settings with limited clinicians. Large language models (LLMs) are DL models pre‐trained on vast amounts of data. LLMs can summarize, translate, and generate content that mimics human language. In their simplest form, LLMs can transcribe physicians' observations and notes through voice‐to‐text technology. They can also support clinical decision‐making, e.g. in the management of multiple sclerosis,^40^ with the ability to demonstrate fluid reasoning in unfamiliar environments.^41^ Current progress is augmenting LLMs with strategies such as supervised fine‐tuning. This grounds LLMs on selected sources, effectively reducing “hallucinations”: LLM texts that are seemingly coherent but are factually incorrect.

#### 4.1.3 AI‐powered tools for diagnostic accuracy: Obstetric ultrasound

Antenatal ultrasound is well suited to AI because of its operator dependence and real‐time modality. DL algorithms such as convolutional neural networks (CNNs) can improve image acquisition, view recognition, anatomical labeling and anomaly detection, and standardization of readouts without replacing expert reviews. Examples of applications are:

- Acquisition: automated plane/view detection with real‐time feedback; scan completeness metrics.
- Biometry: measurement consistency; flagging discordant trends.
- Placenta and cervix: localization, previa/accreta risk cues; cervical length automation with reliability indices.
- Fetal echocardiography: classification, segmentation, quality scoring, progressing to comprehensive 3D/4D workflows.
- Placental/mass characterization: texture/echogenicity‐based analysis for differentials.

Clinical translation requires multisite external validation, out‐of‐distribution testing, operator‐level analyses, and explicit reporting of failure modes, with human‐in‐the‐loop escalation for safety and accountability. In contrast to other medical sectors, the impact of AI on imaging has had lackluster integration within women's health and studies largely lacked clinical validation across multiple datasets (out of field testing), which is crucial for establishing AI modeling reliability.^42^, ^43^

In short, the growing potential of AI‐powered women's health technology is likely to transform the maternity workforce reshaping traditional roles. The demand will grow not only for clinicians but also for professionals with expertise in data science, ML, and health informatics. This shift necessitates training programs for clinicians to develop a foundational understanding of AI to critically evaluate outputs and maintain human oversight (Box 3).

BOX 3 **Global shortfall of skilled maternity care professionals disproportionately affects low‐ and middle‐income countries.**

- AI cannot replace human empathy, communication, and cultural sensitivity but can augment clinical workforce – as a “digital colleague”.
- Utilizing AI to empower caregivers – enhancing respectful and high‐quality care to all women, regardless of geography or socioeconomic status.
- Human‐centric care will be enabled by AI giving patients a central role as drivers of their own care rather than traditional passive consumers of health delivery.

## 5 FUTURE DIRECTIONS

Effectively incorporating AI into existing clinical workflows is a formidable challenge. Many healthcare institutions rely on legacy operational systems that cannot accommodate AI tools. Successful integration requires large‐scale change management and user training, not to mention the associated financial and environmental costs. The following section future‐casts anticipated events to inform strategies for successful AI implementation in maternity.

### 5.1 Adoption of AI women's health technology will likely be led by China and the USA

**FIGURE 4:**
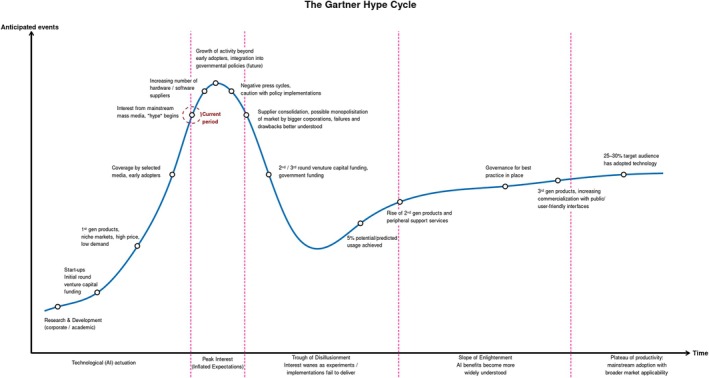
Predicted AI adoption curve based on the Gartner Hype Cycle.

AI adoption is projected to follow an S‐shaped curve (Figure 4) with a “slow burn” start due to significant costs to learn and deploy these technologies, followed by an acceleration from the cumulative effect of competition and improvement in complementary capabilities. The curve for innovation adoption is further dictated by ability to secure funding to cascade from prototypes to scale and sustainability, termed as averting the “valleys of death”.^44^ We are currently in phase 1: the “rise” epoch. To interpret the slow uptake as limitations of AI would be an underestimation of its potential. The benefits for those leveraging these technologies now will build up in later years at the expense of sectors with limited or no adoption.

**FIGURE 5:**
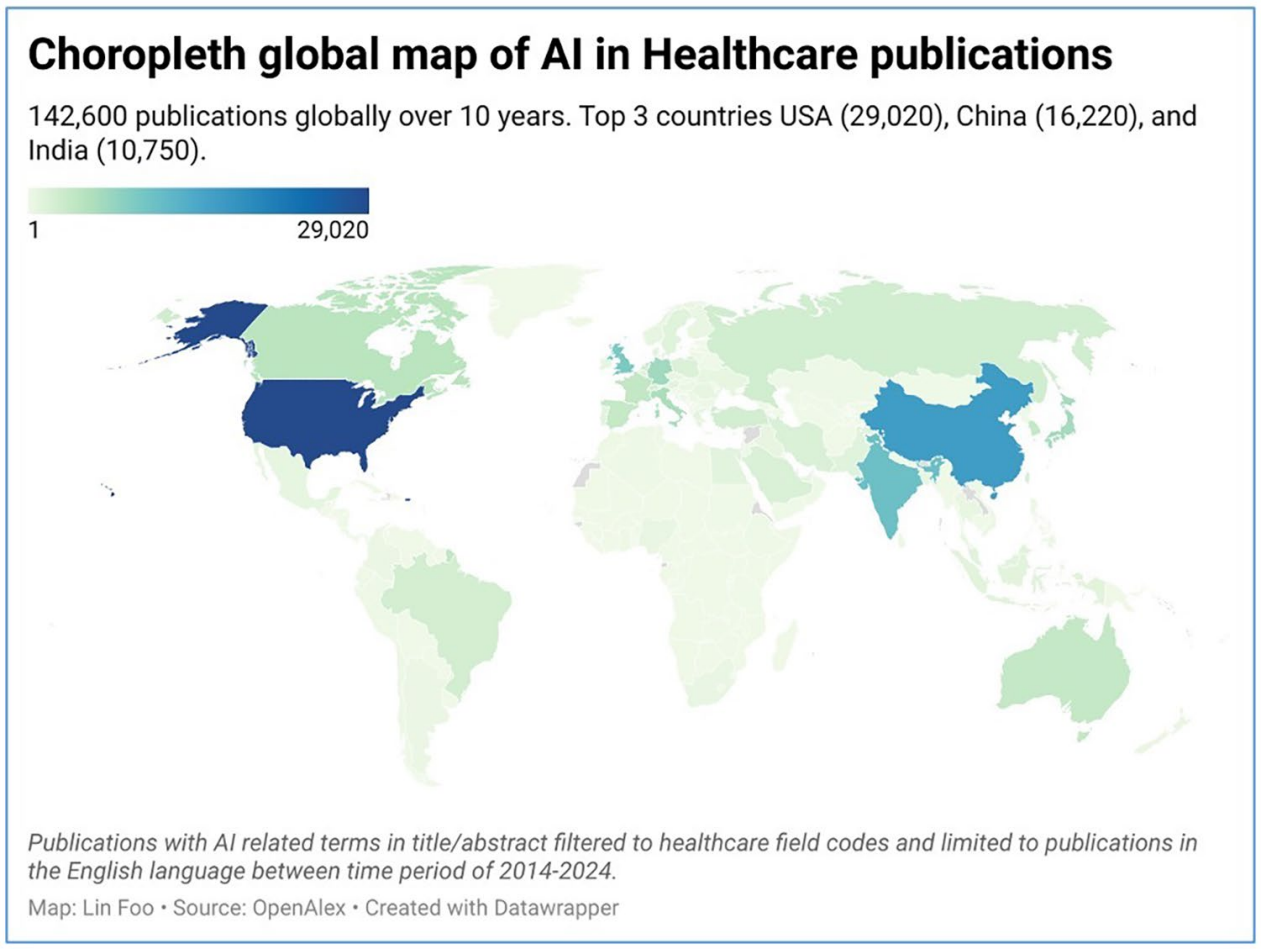
Choropleth map of AI in healthcare‐related publications between 2014 and 2024.

There are a few ways to determine which regions and stakeholders will lead on AI adoptions: surveying (1) influential AI publications, (2) patents, and (3) countries producing the most “notable” AI models. Figure 5 is a choropleth map of publications by countries for AI in healthcare over a decade, scoping articles with field codes ranging from “Healthcare Education” to “Neural dynamics” (LF's own data, Open Alex, accessed June 2025). The USA consistently had the highest number of top‐cited AI healthcare publications, followed by China and India. Sector wise, academic institutions remain the primary source of AI publications, accounting for 84.9% of all AI publications in 2023. In contrast, industry represented 7.1% of all AI publications.^45^ This should not be interpreted as industry having a minor role in AI development; this is perhaps better examined by trends of AI patents.

AI intellectual property patenting is on the rise; between 2010 and 2023, the number of AI patents ballooned from 3833 to 122 511. As of 2023, China leads in total AI patents accounting for almost 70% of all grants.^45^ Data on generative AI patents, one of the most commonly utilized facets in healthcare, recorded 3181 patents from China between 2014 and 2023, compared to 979 from the USA.^46^

Filtering down from patents to meaningful AI models, Epoch AI, an AI Index data provider, utilizes the term “notable ML models” to grade influential models within the AI ecosystem. Nearly 90% of notable models in 2024 (60% in 2023) originated from industry. In 2024, USA‐based companies produced 40 notable AI models, significantly surpassing China's 15 and Europe's combined total of 3.^47^

In short, China and the USA are leaders of the sector, and future models of care implementing AI‐powered women's health technology will likely stem from stakeholders from these regions. The USA and China are the two largest global economies, reflecting the exponential cost of inventing and deploying AI in any sector. However, the above oversimplifies the viewpoint, and factors such as geopolitical changes can influence stakeholders in the field. One such example is regions leading production of crucial hardware such as semi‐conductors. The USA, Japan, and the Netherlands dominate the production of semi‐conductor manufacturing equipment, while Taiwan holds 90% market share of the most advanced chips.^48^ These are key chokepoints and controls over exports could change the long‐term market dominance.

### 5.2 Adoption of AI in higher‐resource countries will eventually reduce costs for all

The cost of training “frontier” AI models (representing the cutting edge of the field) has grown by 2–3 times per year for the last 8 years. By 2027, the largest models will cost over USD 1 billion.^49^ Training costs arise from hardware depreciation and energy consumption, and costs are similar even when alternative approaches, such as cloud training, are adopted. This obviously limits the global reach of AI technologies and paradoxically increases the maternal healthcare gap between high‐ and low‐income countries.

The silver lining comes with economic aging of technology. Moore's Law described exponential computing power increments over time. Fortuitously, technology price changes are not a function of time but of experience. The relationship that each doubling in experience leads to the same relative price decline is described by Wright's Law: due to the learning curve of certain technologies; when more technology is produced, it provides learning opportunities to improve the process. This creates a cycle of increasing demand, more technology gets deployed to satisfy demand, leading to falling prices. At those lower prices, technology becomes cost‐effective in new applications, which, in turn, means that demand increases. From this positive feedback loop, technology powers itself to lower prices.

To examine this effect with AI, we refer to inference costs (the expense of querying a trained AI model, typically measured in USD/million tokens). The cost of querying an AI model that scores the equivalent of ChatGPT 3.5 on Massive Multitask Language Understanding (MMLU), a popular benchmark for assessing LLM performance, dropped from $20.00/million tokens in 2022 to just $0.07/million tokens by 2024, a staggering 280‐fold reduction.^45^ This means that lower‐income countries can have a market share of healthcare–focused AI as the rate of AI developments grow. To further promote equitable AI adoption, scalable and cost‐effective AI solutions, such as open‐source medical AI models and cloud‐based AI platforms, can reduce financial burden while ensuring accessibility.

### 5.3 Strategies for AI implementation in maternity care

The perception among clinicians is of vast potential of AI, but the majority do not know how to leverage this for highest impact.^50^ Best practice has not been established as the field is moving so fast. The following considerations can safeguard transition to clinical settings.

#### 5.3.1 Explainable AI (XAI)

With advanced AI, it can be difficult to retrace how the algorithms produce results, colloquially referred to as “black box” AI. XAI is a set of processes that allows human users to comprehend ML outputs and facilitates accountability by ensuring AI‐led clinical decisions align with ethical, legal, and technical standards. Examples of XAI techniques include agnostic types, such as local interpretable model explanation (LIME) and post‐hoc explanation.^51^ AI algorithms for clinical practice should be published as open access to promote transparency and ease application.

Although every effort should be made to encourage XAI, full implementation may not be realistic for the following reasons. First, explainability relates to the understanding of the algorithms. Although obtainable, the inner workings of AI algorithms may effectively be unintelligible for human understanding. Therefore, clinicians may find themselves liable for AI decisions that are uninterpretable, while remaining incapable of opposing AI if lacking evidence to refute recommendations. Consideration of liability in these situations hinges on the notion that AI tools form only part of comprehensive assessment in decision analysis. One accepts that good decisions can, and do, lead to bad outcomes; this remains true for humans and AI‐enabled decisions alike.^52^ Furthermore, a lack of full scientific understanding of treatment is comparable to current clinical practice; approximately half of guideline recommendations are based on expert‐opinion without higher‐grade supporting evidence.^53^ An example is the use of magnesium sulfate for fetal neuroprotection; the mechanisms of action on the fetal brain remain unclear but do not deter from application when clinical benefits are evident.

Therefore, similar to present‐day clinical practice, liability would consider a clinician's ability to justify their decisions on following or overruling AI recommendations. These are theoretical conjectures as there are currently no specific AI‐healthcare legislative frameworks, with reliance on existing technology‐neutral laws such as data protection to address matters. Forthcoming, a rollout of the EU AI Act, recognized as the world's first comprehensive AI law, should be fully enacted to regulate AI systems, of which two categories—“high‐risk AI” and “AI triggering transparency requirements”—are likely relevant for healthcare.

#### 5.3.2 Standards for maternity‐specific AI

Standards for future women's health‐specific AI training databases should be set to homogenize the quality of AI algorithms. These include data adjudication by experts, standardizing definitions of disease or cutoffs of biomarkers in pregnancy. As we stand at the early stages of AI adoption, it would be prudent to strategize multinational consortia that create and maintain disease‐specific ML benchmark datasets, e.g. “hypertension in pregnancy,” against which future pregnancy specific ML models are validated. ML dataset standards must be balanced by the practicality of adherence in lower‐resource settings.^54^

#### 5.3.3 Legislative frameworks for AI data use

Access to vast amounts of heterogenous data reduces bias in AI algorithms and improves output accuracy. However, maintenance and sharing of large data repositories are vulnerable to data leaks or unauthorized sharing of sensitive health information. There is considerably higher risk if several interfaces are linked, e.g. wearables and IoMT components in agentic AI systems. Currently, there is a lack of AI‐specific data protection laws to govern this, and legislation has to draw from existing frameworks such as the General Data Protection Regulation (2018, EU) and the Health Insurance Portability and Accountability Act (1996, USA).

Future legislative blueprints must consider the following fundamentals: (1) consent for data usage for AI training; (2) algorithmic fairness; and (3) data privacy.^55^ AI data collection involves complex considerations, as individual contributions to systems that make an AI model are often intertwined; thus, it is unclear who truly owns the data. Further, ML‐generated insights introduce further ambiguity of data rights to model intellectual outputs.^56^ Addressing these issues requires collaboration between technology developers, policymakers, field legal experts, clinical users, and patient and public sectors. Stakeholders should plan for approaches such as federated learning (distributed learning where several clients work together on a model but maintain confidentiality of input) or cryptographic techniques to promote the development of AI models with safeguards for data privacy.^57^

#### 5.3.4 Deployment in LMICs

The uneven pace of AI development between high‐income countries and LMICs may lead to the latter occupying consumer positions, rather than innovators or leaders in the future AI sphere. In this, LMICs evolve into “technological colonies” and are subject to AI technology that has not been specifically designed for their population.

The Oxford Insights Government IA Readiness Index^58^ highlights an increasingly bipolar landscape, with the USA and China being dominant forces, and LMICs significantly lagging behind due to major gaps in digital capacity (connectivity, bandwidth limitations), infrastructure (geographical dispersion, technical capacity for electricity, and digital connections), and lack of human resources (data scientists and AI engineers), worsened by “brain drain,” the emigration of talented local AI professionals. Furthermore, low levels of digital literacy with patients remain a present and future obstacle.

Although not an easy task, two key components can elevate the AI preparedness of LMICs, such as strategic government planning and cross‐border efforts.^58^ Government encouragement of local private and public sector collaborations has proven essential in creating new AI ecosystems. An example is the launch of Brazil's AI Sandbox (a controlled testing environment for AI systems), a first in Latin America, which demonstrates successful government‐led commitment to ethical and innovative AI development.^59^ Future roadmaps for executing national‐level AI initiatives may follow a blueprint published by the International Telecommunication Union (ITU).^60^ This consists of analysis and readiness assessment, stakeholder engagement and consultation, national AI strategy drafting, investments for infrastructure, research and development, regulatory frameworks befitting to national interests, capacity and human capital development, public inclusion campaigns, and continuous improvement via monitoring of national rollout projects.

In addition, cross‐border initiatives will help to facilitate technology transfer to countries that struggle to finance AI growth. An example is the United Nations (UN) technology facilitation mechanism (TFM), which serves as a central hub linking countries such as Ethiopia and Serbia to international partners, including the EU and Japan in joint technology initiatives,^61^ while Malaysia and China have agreements on technological exchange (Trusted Data Corridor) and mutual strengthening of AI infrastructure. Indeed, fostering connections between AI global leaders and LMICs could be the impetus for sustainable AI capital growth for the intermediate future.

## 6 CONCLUSION

Despite the potential of AI‐powered technology in obstetrics, caregivers play an irreplaceable role in providing human oversight to foster confidence and morale in caregivers and patients, particularly when AI models become more autonomous. The opacity of these models and scale at which they operate makes real‐time human monitoring challenging.^62^ This highlights the need for focus on designs that incorporate hybrid medical oversight mechanisms, safety‐net based interventions and regulatory compliance.

There is a danger that by upscaling reliance on AI, future maternity caregivers lose non‐technical skills like the ability for clinical reasoning. Close collaboration between clinicians and industry is essential to develop digital solutions that enhance clinical practice without compromising human aspects of care, essentially utilizing AI to augment rather than replace human caregivers. The goal is to leverage AI solutions to strengthen the care and shift obstetrics models safely to the forefront of an AI‐driven era.

## AUTHOR CONTRIBUTIONS

LF and MC produced the manuscript. FIGO committee members contributed to conceptualization, reviewing, editing, and final approval for publication.

## FUNDING INFORMATION

No funding was received for this study.

## CONFLICT OF INTEREST STATEMENT

The authors have no conflicts of interest.

## Data Availability

Data sharing is not applicable to this article as no new data were created or analyzed in this study.
